# The chemomodulatory effects of glufosfamide on docetaxel cytotoxicity in prostate cancer cells

**DOI:** 10.7717/peerj.2168

**Published:** 2016-06-29

**Authors:** Reem T. Attia, Mai F. Tolba, Ruchit Trivedi, Mariane G. Tadros, Hossam M.M. Arafa, Ashraf B. Abdel-Naim

**Affiliations:** 1 Department of Pharmacology, Toxicology and Biochemistry, Faculty of Pharmacy, Future University in Egypt (FUE), Cairo, Egypt; 2 Biology Department, The School of Sciences and Engineering, The American University in Cairo, New Cairo, Egypt; 3 Department of Pharmacology and Toxicology, Faculty of Pharmacy, Ain Shams University, Cairo, Egypt; 4 Pharmaceutical Sciences, University of Colorado Anschutz Medical Center, Aurora, CO, United States; 5 Department of Pharmacology and Toxicology, Faculty of Pharmacy, Modern University for Technology and Information, Cairo, Egypt

**Keywords:** Prostate cancer, Glufosfamide, Apoptosis, PC-3, LNCaP, Docetaxel

## Abstract

**Background**. Glufosfamide (GLU) is a glucose conjugate of ifosfamide in which isophosphoramide mustard is glycosidically linked to the *β*-D-glucose molecule. Based on GLU structure, it is considered a targeted chemotherapy with fewer side effects. The main objective of the current study is to assess the cytotoxic potential of GLU for the first time in prostate cancer (PC) cells representing different stages of the tumor. Furthermore, this study examined the potential synergistic activity of GLU in combination with docetaxel (DOC).

**Methods**. Two different cell lines were used, LNCaP and PC-3. Concentration-response curves were assessed. The tested groups per cell line were, control, GLU, DOC and combination. Treatment duration was 72 h. Cytotoxicity was assessed using sulforhodamine B (SRB) assay and half maximal inhibitory concentration (IC50) was calculated. Synergy analyses were performed using Calcusyn^®^software. Subsequent mechanistic studies included *β*-glucosidase activity assay, glucose uptake and apoptosis studies, namely annexin V-FITC assay and the protein expression of mitochondrial pathway signals including Bcl-2, Bax, Caspase 9 and 3 were assessed. Data are presented as mean ± SD; comparisons were carried out using one way analysis of variance (ANOVA) followed by Tukey-Kramer’s test for post hoc analysis.

**Results**. GLU induced cytotoxicity in both cell lines in a concentration-dependent manner. The IC50 in PC-3 cells was significantly lower by 19% when compared to that of LNCaP cells. The IC50 of combining both drugs showed comparable effect to DOC in PC-3 but was tremendously lowered by 49% compared to the same group in LNCaP cell line. *β*-glucosidase activity was higher in LNCaP by about 67% compared to that determined in PC-3 cells while the glucose uptake in PC-3 cells was almost 2 folds that found in LNCaP cells. These results were directly correlated to the efficacy of GLU in each cell line. Treatment of PC cells with GLU as single agent or in combination with DOC induced significantly higher apoptosis as evidenced by Annexin V-staining. Apoptosis was significantly increased in combination group by 4.9 folds and by 2.1 Folds when compared to control in LNCaP cells and PC-3 cells; respectively. Similarly, the expression of Bcl-2 was significantly decreased while Bax, caspase 9 and 3 were significantly increased in the combined treatment groups compared to the control.

**Conclusion**. GLU has a synergistic effect in combination with DOC as it increases the cell kill which can be attributed at least partially to apoptosis in both the tested cell lines and it is suggested as a new combination regimen to be considered in the treatment of the prostate cancer. Further experiments and clinical investigations are needed for assessment of that regimen.

## Introduction

Prostate cancer (PC) is among the most frequently diagnosed solid tumors in men aside from skin cancer. PC is the second leading cause of cancer death in American men, behind only lung cancer. The public health burden of prostate cancer is outspread. A total of 180,890 new cases of prostate cancer and 26,120 deaths from the disease are anticipated in the United States in 2016. Being old, African man with a family member with PC are all considered risk factors for PC (Pienta & Esper, 1993). Statistics on global cancer burden in 2013 indicated that 1.4 million cases of prostate cancer was reported and thus it was the leading cause for cancer incidence in men (Fitzmaurice et al., 2015).

DOC is a semi-synthetic taxane analogue from the European yew (Taxus baccata) (Gligorov & Lotz, 2004). DOC is a widely known anticancer which acts as a microtubule stabilizer (Mackler & Pienta, 2005). The mechanisms of action of DOC include disrupting the function of microtubules leading to cell cycle arrest at mitosis (Baker et al., 2009). Moreover, it phosphorylates Bcl-2, leading to its inactivation and to eventual cell death by apoptosis (Petrylak et al., 2004). Glufosfamide (GLU) is considered a new generation oxazaphosphorine drug (Mazur et al., 2012). Oxazaphosphorines are a class of bi-functional alkylating agents that have been extensively investigated for their anticancer and immune-regulating activities. GLU was the first cytotoxic glycoconjugate to be synthesized by conjugating glucose with the anti-cancer isophosphoramide mustard (IPM); the active metabolite of ifosfamide (Pohl et al., 1995). Glufosfamide has few side effects because it is a cytotoxic alkylating agent linked to a glucose moiety which help its transport to cancer cells were it is activated (Workman, 1997). Malignant cells are fast dividing cells which use more glucose than normal cells (Seker et al., 2000). Several decades ago, Otto Warburg described this phenomenon whereby cancer cells avidly take up glucose and produce lactic acid under aerobic conditions, a process subsequently referred to as the Warburg effect or aerobic glycolysis (Warburg, 1956). That is the rationale behind one of the cancer drug targeting modalities. The concept of glycoconjugate depends on integrating an anticancer drug with a sugar moiety which will be transported through glucose transporters to their specific binding sites in the tumor cells, where they are selectively up taken and thus, minimizing the organ toxicity of these compounds. In fact, DOC-based chemotherapy improved survival in patients with castration-resistant prostate cancer (CRPC) (Festuccia et al., 2009). Therefore, it has been approved as a first line treatment for this setting. However, its use as monotherapy is accompanied by toxic reactions. The main objective of the current study is to assess the cytotoxic potential of GLU single treatment and in combination with DOC in PC cells; both androgen dependent (LNCaP) and androgen independent (PC-3) cell lines. Moreover, the underlying mechanisms for the possible cytotoxic effects are addressed particularly with regard to apoptosis. Also, the correlation between the cytotoxic potential of GLU and *β*-glucosidase activity and glucose uptake in each cell line is also investigated.

## Materials and Methods

### Chemicals and drugs

GLU was obtained from Threshold Pharmaceuticals (San Francisco, CA, USA). DOC was purchased from LC labs (Woburn, MA, USA). Sulforhodamine B sodium salt (SRB) dye was purchased from Sigma-Aldrich (St. Louis, MO, USA). Cell culture media, fetal bovine serum (FBS), Penicillin/streptomycin solution, were obtained from Invitrogen (Grand Island, NY, USA).

### Cell culture

Human androgen-independent PC cell line PC-3 and human androgen-dependent PC cell line LNCaP were provided by the University of Colorado Cancer Center (Anschutz campus, CO, USA). Human brain cancer cells U87 MG cell line (ATCC^®^ HTB14™) (ATCC, Manassas, VA, USA) was also used. Cells were maintained as described previously (Tolba et al., 2013).

### Cytotoxicity assay and synergy analysis

DOC was dissolved in DMSO and kept at a stock concentration of 100 mM, while GLU was dissolved in PBS and kept at a stock concentration of 100 mM. Single-drug concentration–response curves were assessed. Seeding was done at a density of 2,000 cells/well for PC-3 and LNCaP, in 96-well plates. Cells were treated with each single drug and their combination for 72 h at different drug concentrations. DOC was used at concentrations of 0.1–1,000 nM. GLU was used at concentrations of 0.1–300 µm. Cytotoxicity was assessed at the end of drug exposure using SRB assay as previously described (Skehan et al., 1990). Following 72 h exposure the cells were fixed with 10% trichloroacetic acid (150 µl) for 1 h at 4 °C. Then, cells were stained for 10 min at room temperature with 0.4% SRB dissolved in 1% acetic acid. The plates were then air dried for 24 h and the dye was made soluble with 150 µl Tris (10 mM, PH 7.4) for 5 min on a shaker at 1,600 rpm. Absorbance was then measured at 545 nM using microplate reader. Results were expressed as the relative percentage of absorbance compared to control. The experiments were performed in triplicates (*n* = 3) and each time with six replicates (six wells of the 96 well plate per experimental condition). The half maximal inhibitory concentration (IC50) was calculated using Graphpad Prism 5 software (San Diego, CA, USA). Drug interactions were analyzed by Calcusyn program version 2.1 (Biosoft, Cambridge, UK) based on the analytical method of Chou and Talalay (Chou & Talalay, 1984).

### Fluorescence based-glucose uptake assay

In the glucose related studies U87 MG was chosen to compare its glucose uptake and *β* glucosidase activity to that of the PC cells. The cells were seeded in 96 wells plate with black walls and clear bottom at a density of 3 × 10^4^ cells/well as described (Park, 1999). Glucose uptake was measured using Glucose Uptake Cell-Based Assay Kit (Cayman Chemical, Ann Arbor, MI, USA) according to the manufacturer’s instructions.

### Beta glucosidase activity assay

The activity of *β* glucosidase enzyme was assessed using beta glucosidase assay kit (Abnova, Walnut, CA, USA) according to the manufacturer’s instructions and the *β* glucosidase activity is calculated as described (Bhat, Gaikwad & Maheshwari, 1993; Chadwick et al., 1995).

#### Annexin V FITC/PI apoptosis assay

Apoptosis assay was done using Annexin V-FITC/PI apoptosis assay Kit by BD Biosciences (San Jose, CA, USA) as described previously (Tolba et al., 2013).

### Western blot analysis

Western blotting was performed as described previously by Tolba et al. (2013). Antibodies for Bax, Bcl-2, caspase 9, and caspase 3 were purchased from Cell Signaling Inc, (Danvers, MA, USA) and were used in the ratio of (1:1000).

#### Statistical analysis

Data are presented as mean ± SD; comparisons were carried out using one way analysis of variance (ANOVA) followed by Tukey-Kramer’s test for post hoc analysis. Statistical significance was acceptable to a level of *P* < 0.001. All statistical analysis was performed using Graph pad InStat software, version 3.05 (La Jolla, CA, USA).

## Results

### GLU and DOC combination showed enhanced cytotoxicity in prostate cancer cells

**Figure 1:**
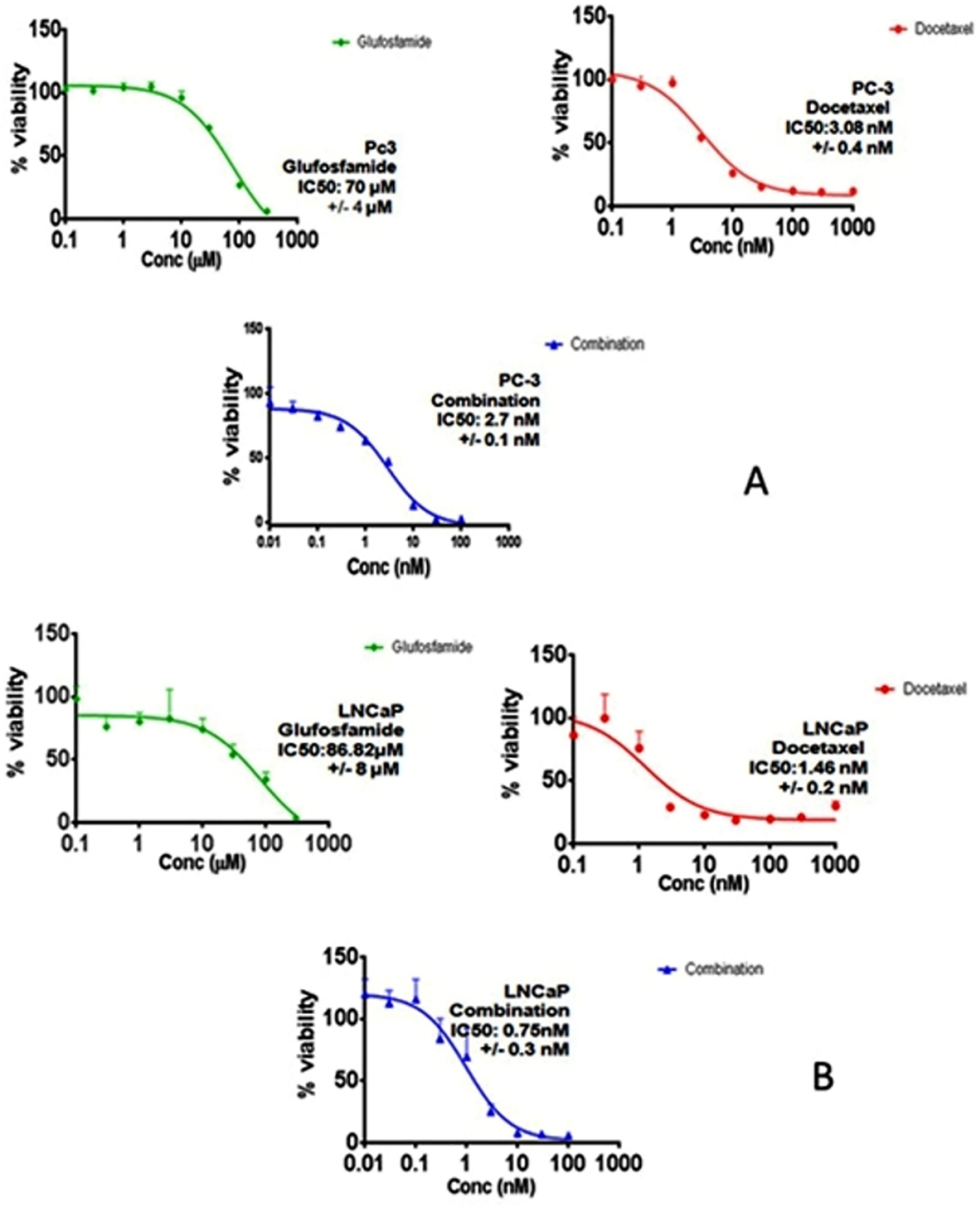
Concentration response curves. (A) The effect of Glufosfamide, Docetaxel, combination on PC-3 cells. (B) The effect of Glufosfamide, Docetaxel, combination on LNCaP cells. Data are means ± SD (*n* = 6). Experiments were done in triplicates.

**Figure 2:**
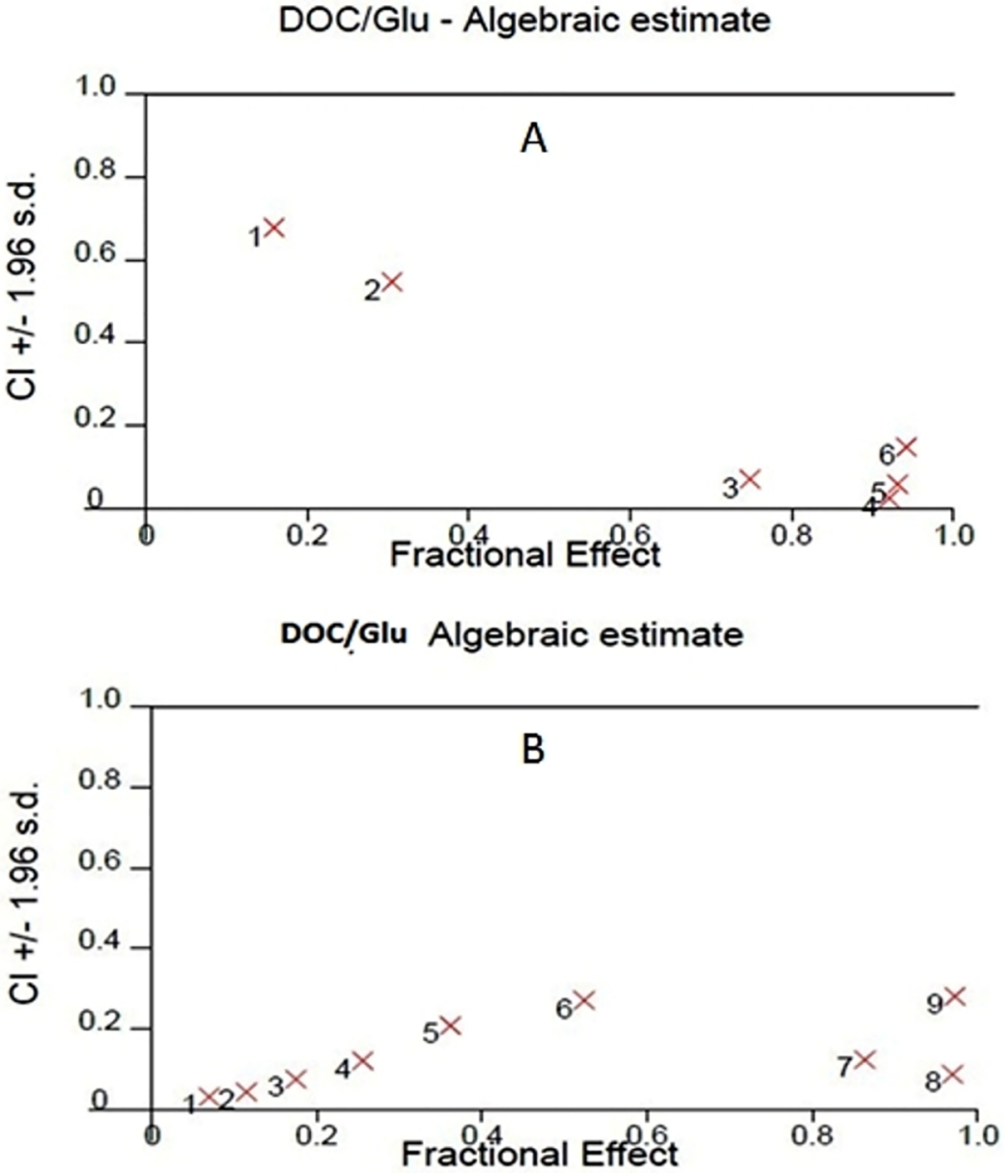
Synergy analysis curve for GLU/DOC combinations in (A) LNCaP PC cells, (B) PC-3 cells. Graph was plotted using calcusyn software.

In order to investigate the effect of GLU, DOC and their combination, concentration–response curves of each drug as single agent were assessed and compared to those obtained from combining the two agents. SRB assay was performed as described before (Skehan et al., 1990) and the concentration–response curves were plotted in both PC-3 and LNCaP. DOC and GLU single and combined treatments affected the cells viability in a dose-dependent manner. The half maximal inhibitory concentrations (IC50) of GLU were 70 ± 4 µM and 86.8 ± 8 µM in PC-3 and LNCaP cells; respectively. The IC50 of GLU was found to be significantly lower in PC-3 by 19% compared to LNCaP. While, the IC50 of DOC alone was found to be 3.08 ± 0.4 nM and 1.46 ± 0.2 nM in PC-3 and LNCaP cells ; respectively. The co-treatment of GLU with DOC was found to synergize the cytotoxicity and the IC50 values were decreased to be 2.7 ± 0.1 nM 0.75 ± 0.3 nM in PC-3 and LNCaP cells; respectively. The concentration–response curve for PC-3 and LNCaP are shown in (Figs. 1A and 1B). The IC50 values of different treatments in all cell lines are shown in Table 1. Synergy analyses were done using Calcusyn software and the combination of GLU/DOC was found to be synergistic in both cell lines as shown in Tables 2 and 3, (Figs. 2A and 2B).

**Table 1:** Inhibitory concentration 50 (IC50) after 72 h treatment for PC-3 and LNCAP cells.

Cell line/drug	GLU	DOC	GLU/DOC
PC-3	70 µM ± 4 µM	3.08 nM ± 0.4 nM	2.7 nM ± 0.1 nM
LNCaP	86.8 µM ± 8 µM	1.46 nM ± 0.2 nM	0.75 nM ± 0.3 nM

**Table 2:** Synergy analysis for GLU/DOC combinations in PC-3 Prostate cancer cells.

GLU/DOC combination			
GLU (nM)	DOC (nM)	Fa	CI
10	0.01	0.069511	0.031
30	0.03	0.114305	0.045
100	0.1	0.17412	0.077
300	0.3	0.255152	0.121
1,000	1	0.361472	0.210
3,000	3	0.523994	0.271
10,000	10	0.863336	0.126
30,000	30	0.970682	0.089
10,0000	100	0.972583	0.282

**Notes.**

TITLE Fa Fraction affected CI Combination index

**Table 3:** Synergy analysis for GLU/DOC combinations in LNCaP Prostate cancer cells.

GLU/DOC combination			
GLU (nM)	DOC (nM)	Fa	CI
300	0.3	0.159245	0.679
1,000	1	0.305415	0.549
3,000	3	0.747996	0.071
10,000	10	0.921549	0.026
30,000	30	0.932034	0.060
10,0000	100	0.942642	0.150

**Notes.**

TITLE Fa Fraction affected CI Combination index

### Glucose uptake in tested PC cell lines

**Figure 3:**
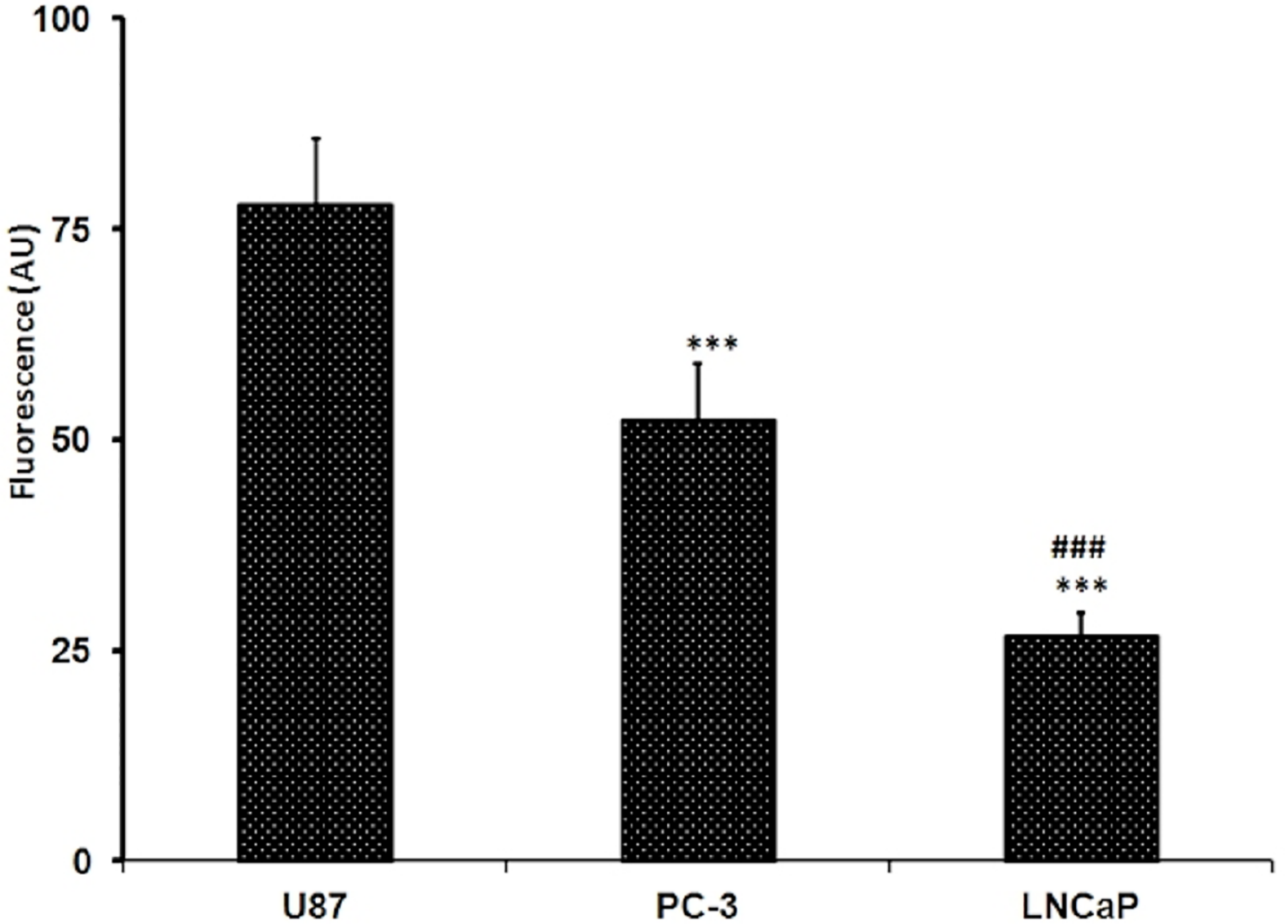
Levels of glucose uptake in U87, PC-3 and LNCaP cell lines. The levels were measured by glucose uptake assay kit using fluorescent glucose 2-NBDG. Data are represented as mean ± SD. (^∗∗∗^) significantly different from U87 at *P* < 0.001. (##) significantly different from PC-3 at *P* < 0.01. Tests were done in triplicates.

The assay was done by fluorometric analysis. Glucose uptake was assessed using fluorescently labeled deoxyglucose analogue 2-NBDG. Fluorescence intensity is directly proportional to the 2-NBDG uptake. PC-3 exhibited significantly higher levels of glucose uptake compared to LNCaP while The U87 MG cell line significantly showed the highest fluorescence absorbance after the 2-NBDG labeling which means that the U87 MG is showing the highest glucose uptake at *p* < 0.001 (Fig. 3). The glucose uptake in U87 MG is amounted to 77.87 ± 7.94 while in PC-3 cells it is amounted to 52.34 ± 6.78, while it was 26.82 ± 2.75 for that detected in LNCaP cells. The glucose uptake in PC-3 cells was almost 2 folds that found in LNCaP cells.

### *β*-Glucosidase activity in tested PC cell lines

**Figure 4:**
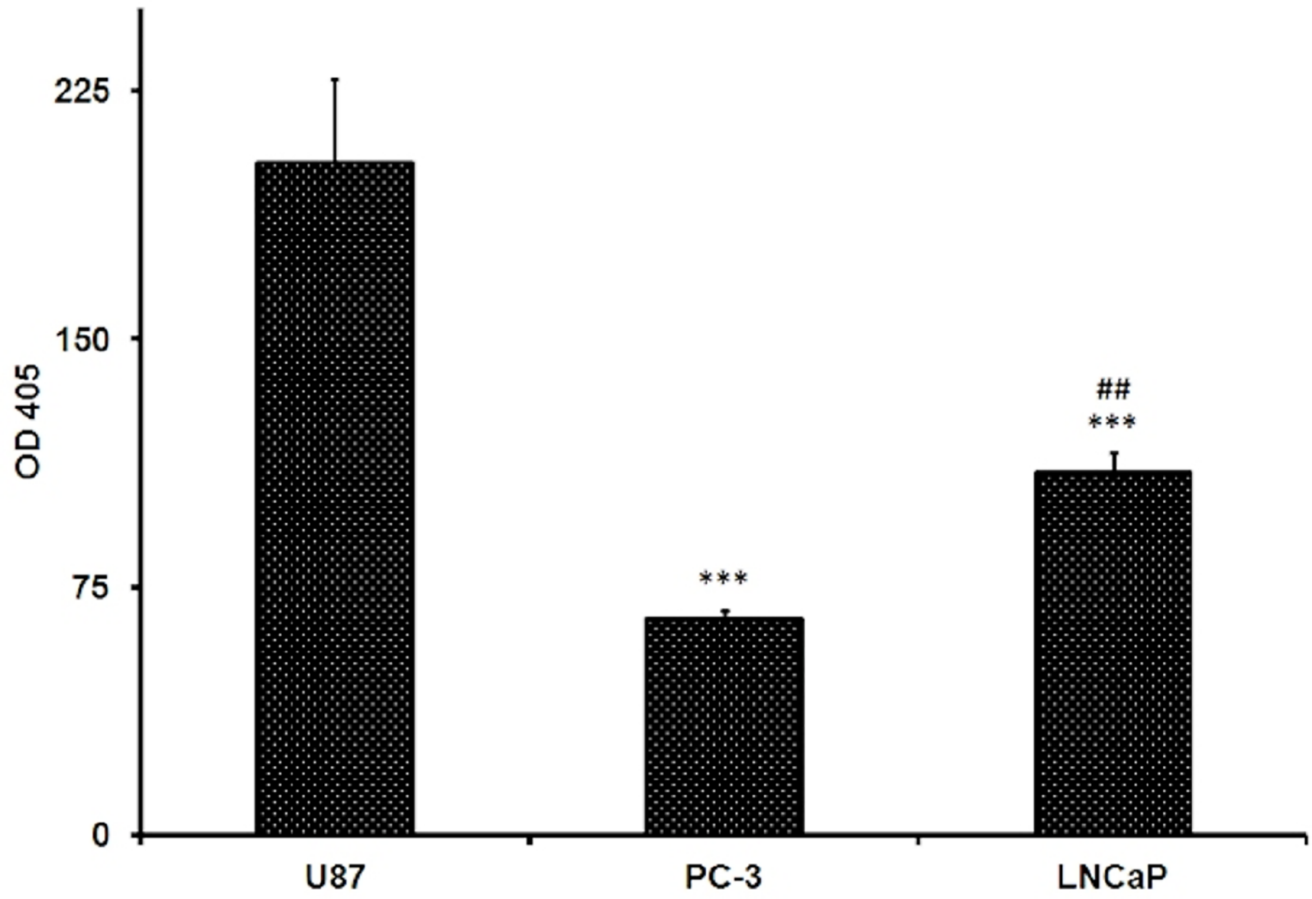
Beta-glucosidase activity in U87, PC-3 and LNCaP cells measured by beta-glucosidase assay kit using the 2-NPG substrate. Data are represented as mean ± SD. (^∗∗∗^) significantly different from U87 at *P* < 0.001. (##) signifcantly different from PC-3 at *P* < 0.01.

Assessment of *β*-glucosidase activity in the cell lysates of both prostate cancer cell lines and U87 cells revealed that the enzyme activity was the highest in U87 cells 203.2 ± 25.3. For prostate cells it was 65.55 ± 2.3 and 109.75 ± 5.8 (U/L) for PC-3 and LNCaP cells, respectively (Fig. 4). It was apparent that the *β*-glucosidase activity was higher in LNCaP by about 67% compared to that determined in PC-3 cells (Fig. 4). The differences were found to be significant at *p* < 0.01. The test was done in triplicates.

**Figure 5:**
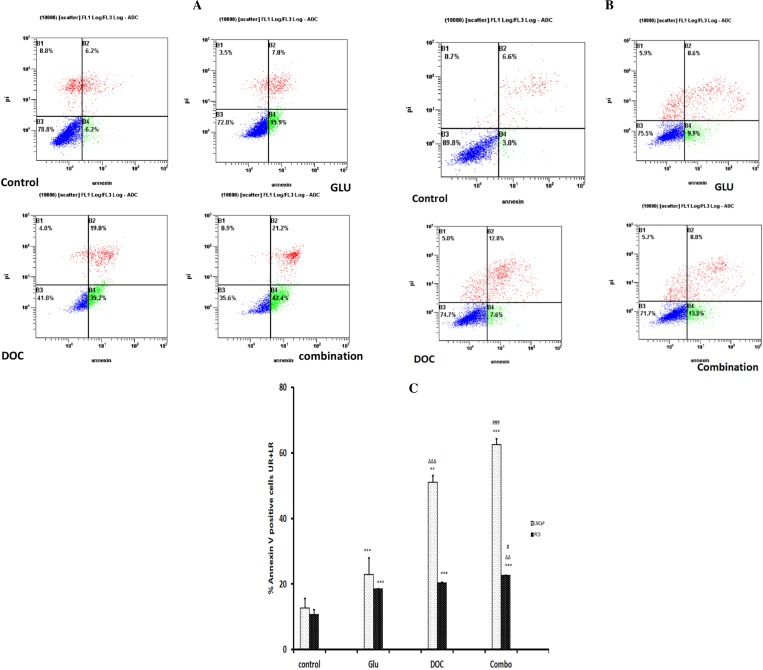
(A) AnnexinV-FITC apoptosis assay for LNCaP cells after treatment for 72 h. The experiment was done in triplicates and a representative figure was chosen for the dot plot. (B) AnnexinV-FITC apoptosis assay for PC-3 cells after treatment for 72 h the experiment was done in triplicates and a representative figure was chosen for the dot plot. (C) Effect of different treatments on the Annexin V-FITC Positive staining in PC-3 and LNCaP Cells. The experiment was done in triplicates. (^∗∗∗^) significantly different compared to the control at *P* < 0.001. (^∗∗^) significantly different compared to the control at *P* < 0.01.). ($\mrm{\Delta }\mrm{\Delta }\mrm{\Delta }$) significantly different compared to GLU at *P* < 0.001. ($\mrm{\Delta }\mrm{\Delta }$) significantly different compared to GLU at *P* < 0.01. (###) Significantly different than DOC at *P* < 0.001. (#) Significantly different than DOC at *P* < 0.05. Data are means ± SD (*n* = 3). Data are means ± SD (*n* = 3).

### GLU/DOC combination significantly increased the percentage of Annexin V-FITC positive cells

Annexin V-FITC/PI apoptosis assay showed that the treatment of LNCaP cells with the combination of DOC and GLU caused a significant increase in the percentage of AnnexinV-FITC positive cells by 4.9 folds when compared to the control (Figs. 5A and 5C). In addition to that, the same combination showed significant increase in the cells labeled with AnnexinV-FITC in PC-3 cells by 2.1 Folds compared to the control (Figs. 5B and 5C).

### GLU/DOC combination altered the expression of Bax, Bcl-2, caspase-9 and caspase-3 in PC-3 and LNCaP

**Figure 6:**
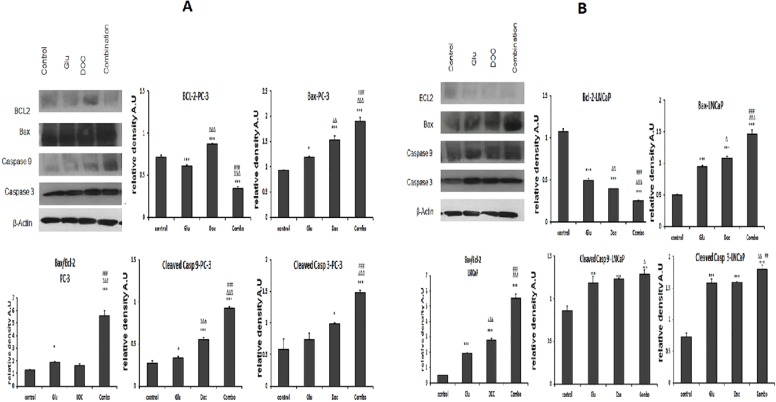
(A) The effect of GLU, DOC and their combination on the expression of mitochondrial apoptosis signaling proteins in PC-3 prostate cancer cells. (^∗^) Significantly different than the control at *P* < 0.05 (^∗∗^) significantly different compared to the control at *P* < 0.01. (^∗∗∗^) significantly different compared to the control at *P* < 0.001. ($\mrm{\Delta }$) significantly different compared to GLU at *P* < 0.05. ($\mrm{\Delta }\mrm{\Delta }$) significantly different compared to GLU at *P* < 0.01. ($\mrm{\Delta }\mrm{\Delta }\mrm{\Delta }$) significantly different compared to GLU at *P* < 0.001. (#) Significantly different than DOC at *P* < 0.05. (##) significantly different compared to DOC at *P* < 0.01. (###) significantly different compared to DOC at *P* < 0.00. Data are means ± SD (*n* = 3) of relative band densities normalized to *β*-actin content. (B) The effect of GLU, DOC and their combination on the expression of many cell survival and apoptotic pathways in LNCaP prostate cancer cells. (^∗^) Significantly different than the control at *P* < 0.05 (^∗∗^) significantly different from the control at *P* < 0.01. (^∗∗∗^) significantly different compared to the control at *P* < 0.001. ($\mrm{\Delta }$) significantly different compared to GLU at *P* < 0.05. ($\mrm{\Delta }\mrm{\Delta }$) significantly different compared to GLU at *P* < 0.01. ($\mrm{\Delta }\mrm{\Delta }\mrm{\Delta }$) significantly different compared to GLU at *P* < 0.001. (#) Significantly different than DOC at *P* < 0.05. (##) significantly different compared to DOC at *P* < 0.01. (###) significantly different compared to DOC at *P* < 0.001. Data are means ± SD (*n* = 3) of relative band densities normalized to *β*-actin content.

The GLU/DOC combination significantly down-regulated the expression of the anti-apoptotic protein Bcl-2 by 75.9% and 55% in LNCaP and PC-3; respectively (Figs. 6bB and 6aB). Moreover, it significantly increased the expression of the pro-apoptotic protein Bax in the two cell lines by 2.8 and 1.9 folds in LNCaP and PC-3 cells; respectively (Figs. 6bC and 6aC). Moreover Bax/Bcl-2 ratio was calculated for both cell lines and there was a significant increase in the ratio in the combination-treated group by about 4.6 and 12 folds in PC-3 and LNCaP cells; respectively compared to the control (Figs. 6aD and 6bD).

The expression level of cleaved caspase 9 and 3 were increased too after the treatment of the tested cells with the GLU/DOC combination. Caspase 3 was significantly increased by 2.3 and 2 folds ( Figs. 6bE and 6aE), while, caspase 9 was significantly increased by 1.5 and 3.2 folds in LNCaP and PC-3 cells; respectively compared to control (Figs. 6bF and 6aF).

## Discussion

PC and particularly its metastatic phenotype, is the second leading cause of death from cancers in American men (Siegel, Miller & Jemal, 2016). GLU is a glucose conjugate of ifosfamide. The alkylating metabolite of ifosfamide; isophosphoramide mustard (IPM); is linked to the *β*-D-glucose molecule. This moiety coupled to IPM confers drug stabilization and selective uptake of the compound by rapidly dividing cells (Mazur, Opydo-Chanek & Stojak, 2011).

DOC, a taxane used to treat a variety of solid tumors. It has been approved as the first line chemotherapeutic agent for PC since 2004 (Petrylak et al., 2004; Tannock et al., 2004). At present, development of therapies with a multitude of cellular targets has been considered a good strategy to conquer drug resistance and decrease the adverse effects (Yeluri et al., 2009; Calvaresi & Hergenrother, 2013). Thus, the lack of selectivity of cytotoxic drugs and the development of multidrug resistance have given impulse to the development of target-specific new compounds (Wu, Calcagno & Ambudkar, 2008).

Although, DOC is considered the first line treatment for the metastatic castration resistant prostate cancer (CRPC), the high-dose of DOC as monotherapy is associated with significant toxic effects (Obasaju & Hudes, 2001). For that reason, many therapeutic interventions are currently on the run with the aim of improving the therapeutic index of DOC-based chemotherapy (Tse et al., 2014). This study was conducted in order to assess cytotoxic potential of GLU in PC cells. In addition to that, it defines the underlying mechanisms for the possible cytotoxic potential with emphasis on apoptosis. Moreover, it sheds a light over the correlation between cytotoxic potential of the GLU, the beta-glucosidase activity and glucose uptake in PC cell lines, and to evaluate the effect of GLU on DOC cytotoxicity.

Drug combination is most widely used in treating the most dreadful diseases, such as cancer and AIDS. The main aims are to achieve synergistic therapeutic effect, to fulfill dose and toxicity reduction, and to minimize or delay the induction of drug resistance (Chao Chou, 2010). Recent drug discovery and development studies are focused on maximizing the therapeutic efficacy of potent anti-cancer drugs at low dose levels (Farrell et al., 2011). The biology of PC evolves from a small, slow-growing, androgen dependent carcinoma toward a more and more aggressive, androgen independent tumor during the course of progression (Chen et al., 2004). This is in line with our findings in the current study. Cytotoxicity assay was performed on the two PC cell lines; PC-3 (androgen-independent) and LNCaP (androgen-independent). Our findings indicated that the androgen-independent PC-3 cells showed glucose uptake more than that of the less aggressive LNCaP which was obviously related to our cytotoxicity results. GLU exhibited concentration-dependent cytotoxicity with IC50 70 µM and 86.8 µM in PC-3 and LNCaP cells; respectively. Interestingly, it was found that potency of GLU was significantly higher in the advanced stage-IV PC-3 cells compared to LNCaP. On the other hand, DOC single treatment showed cytotoxicity with IC50 1.46 nM and 3.08 nM in LNCaP and PC-3 cells, respectively. These results were in-line with another study that showed IC50 of DOC in PC-3 cells to be 3.9 nM (Pinto, Moreira & Simoes, 2009). The combined treatment of GLU and DOC reduced IC50 to 0.75 nM and 2.7 nM in LNCaP and PC-3 cells; respectively. The results showed that the combination was more effective in LNCaP cells as evidenced by reduction in DOC IC50 significantly by 51% compared to DOC single treatment.

These cytotoxicity results raised the need for more investigations. Therefore, we assessed the difference in the uptake and hydrolysis of the GLU in both cell lines. Stage IV glioblastoma multiform U87 MG cells are known for their elevated glycolysis and glucose uptake (Beckner et al., 2005; Beckner et al., 2009). Moreover, *in vivo* experiments performed on intact human brain tumors demonstrated that their metabolism involves extensive glucose oxidation (Maher et al., 2012) and that these cells utilize mostly glucose and glutamine for nutrition (Marin-Valencia et al., 2012). Thus, U87 MG was chosen as a positive control to compare its glucose uptake and -glucosidase activity to the prostate cancer cells tested. GLU cytotoxicity was assessed in the U87 MG cells and it showed highly significant increase in potency of GLU against this aggressive tumor (R Attia, pers. comm., 2016). U87 MG cells showed the highest *β*-glucosidase activity. Interestingly, it was found that *β*-glucosidase activity in LNCaP cells is 67% significantly higher than that in PC-3 cells, with 49% decrease and 68% decrease compared to U87 MG in LNCaP and PC-3; respectively.

This observation was contradictory to the IC50 values computed for both cell lines let for other previously reported data on GLU. Seker et al. (2000) concluded by using computer-assisted automated cell microinjection, that GLU induced the highest cytotoxicity in MCF-7 more than LLC-PK1 and Nalm-6 cells and this significantly correlated with the observed elevated *β*-glucosidase activity in this breast cancer cell line. Additionally, Arafa (2009) reported that the cytotoxicity rank order of GLU in colorectal cancer cell lines; Caco-2, HT29 and H84, was well correlated with the *β*-glucosidase enzymatic activities in all cell lines. GLU has been identified as a substrate for cytosolic and lysosomal *β*-glucosidases (Arafa, 2009; Seker, Bertram & Wießler, 1996) this is considered as a strong indication for the importance of this enzyme in the activation of the glycoconjugate.

U87 MG cells significantly showed the highest glucose uptake, while glucose uptake was found to be significantly higher in PC-3 cells by nearly 2 folds compared to LNCaP cells, with 33% decrease and 65% decrease compared to U87 MG cells in PC-3 and LNCaP cells; respectively. The higher glucose uptake in PC-3 is in-line with previous data indicating that glycolysis is higher in aggressive tumors (Lacombe, 2012). Thus, one could argue that despite the importance of the intracellular glucosidases in tumor cells, however, glucose uptake is a crucial rate-limiting step in GLU cytotoxicity. The earlier notion that high cellular *β*-glucosidase levels seem only to be effective when transport proteins are expressed as well, may lend support to this view (Nomoto et al., 1999). Consistent with that was the finding that spontaneous hydrolysis of GLU into its IPM aglycone occurred in various biological samples and *β*-glucosidase had a negligible effect (Sun et al., 2006).

The combination of GLU/DOC resulted in a promising cytotoxicity in PC. Therefore, the current study was further substantiated in order to investigate the underlying mechanisms. Since induction of apoptosis is an important mechanism of cytotoxicity of both DOC (Mackler & Pienta, 2005) and GLU (Zhang, Tian & Zhou, 2006). Initially Annexin V FITC apoptosis assay was done. Treatment of the cells with GLU/DOC induced significant increase in the percent of Annexin V positive apoptotic cells by about 62.5% and 22.6% in LNCaP and PC-3; respectively when compared to the control. GLU was previously reported to induce apoptosis in L1210 leukemia cells. It was also effective in Erlich ascites carcinoma and P388 leukemia in mice, and L5222 leukemia (Pohl et al., 1995). Moreover, it was effective in breast carcinoma cells, MCF-7 (Seker et al., 2000) and childhood acute leukemic lymphoblast. Similarly, DOC showed pro-apoptotic effects as an adjuvant chemotherapy for early-stage breast cancer (Piccart, 2003). Moreover it was effective against non-small cell lung cancer (NSCLC) (Rapp et al., 1988; Non-Small Cell-Lung-Cancer Collaborative-Group, 1995), refractory solid tumors in pediatric patients (Seibel et al., 1999) and prostate cancer cells (Tannock et al., 2004).

Bcl-2 family members are important in the regulation and control of the intrinsic apoptosis pathway. In the current study, the expression of Bcl-2 was significantly decreased on treatment with the combination compared to the control in both tested cell lines. DOC was reported to induce down-regulation of Bcl-2 in C4-2B PC cell line (Yoo, Park & Lee, 2008) and DU145 cells (Haldar, Chintapalli & Croce, 1996). However, in the current study DOC didn’t show a significant decrease in Bcl-2 expression in PC-3 cells which came in-line with other studies in which Bcl-2 phosphorylation and expression was not significantly altered on treatment with DOC in PC-3 cells (Ting et al., 2007). In addition to other studies suggesting that DOC sensitizes PC-3 apoptosis induced by TRAIL but does not induce significant changes in the intracellular levels of Bcl-2 (Yoo, Park & Lee, 2008; Freitas et al., 2011). GLU was previously shown to induce down-regulation of Bcl-2 in V79B and CL-V5B cells (Becker et al., 2002). Bax is another important Bcl-2 family member which opposes the action of Bcl-2 leading to activation of the caspase cascade reaction, eventually resulting in apoptosis (Wang et al., 2002; Ji & Ji, 2014). In the current study, GLU/DOC combination showed significant increase in the Bax expression in PC-3 and LNCaP cells when compared to the control. These results were in-line with other studies in which cells treated with DOC showed significant increase in Bax including LNCaP and PC-3 (Ting et al., 2007). In addition to lymphoid cells (Haldar, Jena & Croce, 1995). It was shown that the Bax/Bcl-2 ratio determines the apoptotic potential of a cell (Perlman et al., 1999). This means that a cell with a high Bax/Bcl-2 ratio will be more sensitive to a given apoptotic stimulus when compared to a similar cell type with a comparatively low Bax/Bcl-2 ratio (Perlman et al., 1999). In the current study, Bax/Bcl-2 ratio was calculated for each treated group and the highest Bax/Bcl-2 ratio was observed in the combination group which was significantly increased in both tested cell lines compared to the control. This indicates enhanced sensitivity to apoptosis after the combined GLU/DOC treatment.

The subsequent step was testing the effect of the different treatments on caspase-9 and caspase-3. Caspase-9 is the essential initiator caspase required for apoptosis signaling through the mitochondrial pathway and is activated on the apoptosome complex (Wurstle, Laussmann & Rehm, 2012). Caspase-3 is known as a cysteine protease that executes the cell death program (Cheng et al., 2008). In the current study, the combined treatment with GLU/DOC boosted the expression levels of cleaved caspase-9 and cleaved caspase-3 compared to control in PC-3 and LNCaP cells; respectively. Previous studies indicated that GLU was found to significantly increase the activity of caspase-9 and -3 in colon cancer cell lines; Caco-2, NHT29 and NT84 (Arafa, 2009) and in Chinese hamster cells, CL-V5B (Becker et al., 2002). Similarly, DOC was found to increase the caspase-3 activity and protein abundance in PC cells; PC-3 (Tolba et al., 2013) and ovarian cancer cells; OVCAR-3(Geertruida et al., 2002).

In the current study, PC-3 cells were less sensitive to the drug than LNCaP. Similar finding was recently reported (Tamaki et al., 2014). No data are available so far on the cytotoxic effects of DOC plus any oxazaphosphorine in prostate cancer except for the report of Cardillo et al. (2013) ifosfamide was found to have only minimal overlapping non-hematologic toxicity. Recalling that GLU has shown short-lived neutropenia or leucopenia in advanced solid tumor patients (Niculescu-Duvaz, 2002), so likewise, it is likely that the overlapped hematotoxicity of the combo would be minimal when the *in vitro* results are extrapolated from bench to the clinical setting.

## Conclusion

In conclusion, based on these broad observations, one could conclude that the *in vitro* data collected from the current study, when well analyzed, would point out, for the first time, to the oncolytic activity of GLU in two prostate cell lines; namely androgen-dependent LNCaP and androgen-independent PC-3 cells. Glucose uptake was found to be more rate limiting factor for GLU potency in PC cells than *β*-glucosidase. In addition, both drugs have shown synergistic apoptotic effects when combined together through completely diverse mechanisms of action. Finally, the potential merit of GLU/DOC combination warrants further investigations.

##  Supplemental Information

10.7717/peerj.2168/supp-1 Supplemental Information 1 Data S1

## Additional Information and Declarations

### Data Availability

The following information was supplied regarding data availability: The raw data has been supplied as Data S1.
