# Supplementary material for: The chemomodulatory effects of glufosfamide on docetaxel cytotoxicity in prostate cancer cells
Source: PeerJ. 2016 Jun 29;4:e2168. doi: 10.7717/peerj.2168 (PMC4933087; doi:10.7717/peerj.2168)
Supplement: Supplemental Information 1 [file peerj-04-2168-s001.docx]

**Annexin:**

control Glu Doce Combo

Mean LNCaP 12.7 23 51 62.5

PC3 10.7 18.5 20.4 22.6

control Glu Doce Combo

SD LNCaP 2.9 5.06 2.07 1.9

PC3 1.6 0.2 0.3 0.23

**Western blot:**

1-PC-3:

| Bcl-2 | Mean | SD | Bax | Mean | SD | Casp 8 | Mean | SD | Casp 9 | Mean | SD | Casp 3 | Mean | SD |
| --- | --- | --- | --- | --- | --- | --- | --- | --- | --- | --- | --- | --- | --- | --- |
| control | 1.04 | 0.038325 | control | 0.49 | 0.010797 | control | 1.15 | 0.20036523 | control | 0.79 | 0.056998853 | control | 0.80 | 0.064851 |
| Glu | 0.49 | 0.022177 | Glu | 0.94 | 0.017 | Glu | 0.95 | 0.12324458 | Glu | 1.09 | 0.082201203 | Glu | 1.56 | 0.064788 |
| Doc | 0.39 | 0.00218 | Doc | 1.09 | 0.035366 | Doc | 1.71 | 0.03847915 | Doc | 1.22 | 0.009906751 | Doc | 1.60 | 0.012586 |
| combo | 0.25 | 0.003402 | combo | 1.41 | 0.074697 | combo | 1.33 | 0.0576111 | combo | 1.25 | 0.053378333 | combo | 1.86 | 0.070136 |

2-LNCaP:

| Bcl-2 | Mean | SD | Bax | Mean | SD | Casp 8 | Mean | SD | Casp 9 | Mean | SD | Casp 3 | Mean | SD |
| --- | --- | --- | --- | --- | --- | --- | --- | --- | --- | --- | --- | --- | --- | --- |
| control | 1.04 | 0.038325 | control | 0.49 | 0.010797 | control | 1.15 | 0.20036523 | control | 0.79 | 0.056998853 | control | 0.80 | 0.064851 |
| Glu | 0.49 | 0.022177 | Glu | 0.94 | 0.017 | Glu | 0.95 | 0.12324458 | Glu | 1.09 | 0.082201203 | Glu | 1.56 | 0.064788 |
| Doc | 0.39 | 0.00218 | Doc | 1.09 | 0.035366 | Doc | 1.71 | 0.03847915 | Doc | 1.22 | 0.009906751 | Doc | 1.60 | 0.012586 |
| combo | 0.25 | 0.003402 | combo | 1.41 | 0.074697 | combo | 1.33 | 0.0576111 | combo | 1.25 | 0.053378333 | combo | 1.86 | 0.070136 |

**Betaglucosidase assay:**

|  | 1 | 2 | 3 | Mean | SD |
| --- | --- | --- | --- | --- | --- |
| U87 | 226.8012 | 206.476 | 176.3387 | 203.2053 | 25.38978 |
| LNCAP | 114.2417 | 111.8587 | 103.1679 | 109.7561 | 5.828602 |
| PC3 | 63.3 | 65.3 | 68.05 | 65.55 | 2.384848 |
|  |  |  |  |  |  |

**Glucose uptake:**

|  |  |  |  |  | average | SD |
| --- | --- | --- | --- | --- | --- | --- |
| u87 | 89.489 | 76.399 | 73.564 | 72.049 | 77.87525 | 7.949645 |
| pc3 | 50.257 | 55.995 | 59.28 | 43.836 | 52.342 | 6.78674 |
| Lncap | 25.724 | 29.473 | 28.64 | 23.478 | 26.82875 | 2.75201 |

**IC50:**

1-PC-3:

**GLU:**

|  | #1 | #2 | #3 | Average | SD |
| --- | --- | --- | --- | --- | --- |
| IC_50_ | 74.66 | 67.1 | 68 | 69.92 | 4.129552 |

**DOC:**

|  | #1 | #2 | #3 | Average | SD |
| --- | --- | --- | --- | --- | --- |
| IC_50_ | 3.08 | 3.45 | 2.7 | 3.08 | 0.3750111 |
|  |  |  |  |  |  |

**Combination:**

|  | #1 | #2 | #3 | Average | SD |
| --- | --- | --- | --- | --- | --- |
| IC_50_ | 2.87 | 2.78 | 2.63 | 2.76 | 0.1212436 |

2-LNCaP:

**GLU:**

|  | #1 | #2 | #3 | Average | SD |
| --- | --- | --- | --- | --- | --- |
| IC_50_ | 82.3 | 96 | 81.5 | 86.60 | 8.1504601 |

**DOC:**

|  | #1 | #2 | #3 | Average | SD |
| --- | --- | --- | --- | --- | --- |
| IC_50_ | 1.2 | 1.6 | 1,6 | 1.40 | 0.2828427 |

**Combination:**

|  | #1 | #2 | #3 | Average | SD |
| --- | --- | --- | --- | --- | --- |
| IC_50_ | 0.98 | 0.91 | 0.36 | 0.75 | 0.3395585 |
